# Psychosocial interventions for cardiac surgery patients: context of interdisciplinary interaction

**DOI:** 10.1192/j.eurpsy.2024.1025

**Published:** 2024-08-27

**Authors:** O. Nikolaeva, V. Babokin, N. Trofimov, M. Alhasan

**Affiliations:** ^1^Department of Hospital Therapy, Ulianov Chuvash State University; ^2^Chuvash Republic Cardiology Clinic; ^3^Department of Surgical Diseases; ^4^Medical Faculty, Ulianov Chuvash State University, Cheboksary, Russian Federation

## Abstract

**Introduction:**

Multi-staged and personalized in nature, psychosocial interventions for cardiac surgery patients explain the necessity of relying on the potential of interdisciplinary interaction

**Objectives:**

To present the review of the model of interdisciplinary interaction of experts and institutions in the course of psychosocial interventions for cardiac surgery patients, which is currently used in the Chuvash Republic.

**Methods:**

This model involves the experts and institutions of the regional healthcare system, the regional system of social care, the regional and federal system of education, non-governmental medical and health resort institutions, and private practitioners.

**Results:**

In the center of this model is the Regional Cardiology Center, which interacts with the Psychotherapeutic Center and the Republican Mental Hospital’s Helpline. It also involves the town hospitals and the central district hospitals, non-governmental clinics and private practitioners. The process of rehabilitation and follow-up care continues in the regional health resorts. The social service centers provide additional support. The institute of chief experts of the regional Health Care Ministry, which includes a psychiatrist, cardiologist, psychotherapist, and psychologist, oversees the overall activity. The clinic faculty professors and associate professors are involved in the development of the programs.

**Conclusions:**

The practical results of using the regional model of interdisciplinary interaction enhance the potential of psychosocial interventions for cardiac surgery patients.

**Disclosure of Interest:**

None Declared

